# Eukaryotic mRNA Decapping Activation

**DOI:** 10.3389/fgene.2022.832547

**Published:** 2022-03-23

**Authors:** Elva Vidya, Thomas F. Duchaine

**Affiliations:** ^1^ Goodman Cancer Institute, McGill University, Montréal, QC, Canada; ^2^ Department of Biochemistry, McGill University, Montréal, QC, Canada

**Keywords:** mRNA decapping and decay, Dcp1/Dcp2, Edc1, Edc3, Edc4, PatL1, Cup/Me31B/Tral complex, P bodies

## Abstract

The 5**′**-terminal cap is a fundamental determinant of eukaryotic gene expression which facilitates cap-dependent translation and protects mRNAs from exonucleolytic degradation. Enzyme-directed hydrolysis of the cap (decapping) decisively affects mRNA expression and turnover, and is a heavily regulated event. Following the identification of the decapping holoenzyme (Dcp1/2) over two decades ago, numerous studies revealed the complexity of decapping regulation across species and cell types. A conserved set of Dcp1/2-associated proteins, implicated in decapping activation and molecular scaffolding, were identified through genetic and molecular interaction studies, and yet their exact mechanisms of action are only emerging. In this review, we discuss the prevailing models on the roles and assembly of decapping co-factors, with considerations of conservation across species and comparison across physiological contexts. We next discuss the functional convergences of decapping machineries with other RNA-protein complexes in cytoplasmic P bodies and compare current views on their impact on mRNA stability and translation. Lastly, we review the current models of decapping activation and highlight important gaps in our current understanding.

## Introduction

In counteracting gene transcription, mRNA decay largely shapes transcriptome landscapes (Hargrove and Schmidt, 1989; Alkallas et al., 2017). Since the advent of genomics, it became clear that mRNA decay is a major determinant for gene expression, with particularly critical functions in acute developmental transitions (Bashirullah et al., 1999; Vastenhouw et al., 2019), cell division (Krenning et al., 2022) and differentiation (Neff et al., 2012; Battich et al., 2020), in response to external stimuli (Rabani et al., 2011; Kawata et al., 2020), or in viral infection (Abernathy and Glaunsinger, 2015; Guo et al., 2018). While mRNA decay is a regulated series of coordinated molecular events, the “decision” to remove the 5′-cap from an mRNA certainly represents its most critical step (Furuichi et al., 1977; Hsu and Stevens, 1993). Here, we review the current models of decapping activation by outlining the roles of key structural determinants and the molecular functions of decapping activator proteins. We identify and contrast the conserved and diverging features of Dcp2 and Dcp1, as well as known roles and interactions of decapping activators across model species. We then review the apparent convergence of decapping factors in P bodies and discuss its possible functional implications. Finally, we compare the current models of decapping activation in yeast and metazoans and reflect on some of the most important persisting questions.

For consistency, when discussing orthologous genes and proteins that have unrelated names in different species, we use the human nomenclature and indicate species-specific names in brackets.

The 5′-cap structure largely governs the fate and lifespan of eukaryotic mRNAs; it affects activities such as pre-mRNA processing, export, translation, and controls decay by protecting mRNAs from 5′-to-3′ exonucleolytic activities (Topisirovic et al., 2011). The majority of eukaryotic mRNAs (∼88% in human cells) (Wang et al., 2019) are characterized by an N^7^-methylguanosine (m^7^G) cap linked to the first genome-encoded nucleotide via a 5′-5′ triphosphate linkage (Adams and Cory, 1975; Furuichi et al., 1975). The m^7^G cap can also exist in alternative chemical forms (reviewed in Ramanathan et al., 2016; Borbolis and Syntichaki, 2021; Pelletier et al., 2021). For instance, in addition to the minimal m^7^G group (Cap 0), the first and second-in-line nucleotides in most mammalian mRNAs are methylated on the 2′-ribose group (2′-O-methyl ribose), yielding the Cap 1 and Cap 2 structures, respectively (Furuichi, 2015). Additionally, the first adenosine of the transcript can be methylated at the N6 position to produce an m^7^Gpppm^6^Am structure (m^6^Am) that can influence mRNA translation and stability (Mauer et al., 2017; Akichika et al., 2019). A Cap 4 structure, in which the first four encoded nucleotides are methylated, is uniquely found in kinetoplastids such as *Trypanosoma* (Perry et al., 1987), and 2,2,7-trimethylguanosine (TMG) caps are often found in *trans*-spliced mRNAs, which are commonly found in *C. elegans*, and in some noncoding RNAs (Mattaj, 1986; Van Doren and Hirsh, 1990). Non-canonical cap structures such as NAD^+^, FAD^+^ and dephospho-CoA (dpCoA) are also found in a subset of eukaryotic mRNAs, possibly influencing mRNA stability in response to specific metabolic states (reviewed in Kiledjian, 2018; Wiedermannová et al., 2021).

mRNA decapping, the regulated removal of the m^7^G cap, requires hydrolysis of one of the pyrophosphate bonds within the 5′-5′ triphosphate linkage. The Shatkin lab reported the first evidence of RNA decapping by incubating short (7–10 nucleotides long) m^7^G-capped reovirus mRNA in HeLa cell extracts, yielding m^7^GMP and diphosphorylated ribonucleotides (Nuss et al., 1975). This activity was later attributed to the scavenger decapping enzyme DcpS, which functions downstream of the cytoplasmic exosome complex (Wang and Kiledjian, 2001; Liu et al., 2002). Shortly after, Audrey Stevens described a different decapping activity that targets full-length mRNAs from *S. cerevisiae* (Stevens, 1980). It was noted early on that this activity is preceded by poly(A) tail removal/deadenylation and leads to accelerated degradation by the 5′-to-3′ exonuclease Xrn1 (Decker and Parker, 1993; Hsu and Stevens, 1993; Muhlrad et al., 1994). A genetic screen for decapping mutants eventually identified the first decapping co-factor Dcp1, which was originally thought to harbor the catalytic activity (Beelman et al., 1996; Hatfield et al., 1996). Furthermore, a screen for temperature-sensitive suppressors of *dcp1* and *ski8* (a cytoplasmic exosome component) deletions identified a second decapping factor named Dcp2 (previously named Psu1), which provided decapping activity along with Dcp1 (Dunckley and Parker, 1999). It was curious then, that immunoprecipitated Dcp2 had no detectable decapping activity despite bearing the highly conserved pyrophosphohydrolase (Nudix/MutT) motif absent in Dcp1 (Dunckley and Parker, 1999). Subsequently, three independent studies demonstrated that indeed human Dcp2 has an intrinsic decapping activity both *in vitro* and *in vivo* (Lykke-Andersen, 2002; van Dijk et al., 2002; Wang et al., 2002). The Séraphin lab first reported that a C-terminal truncated fragment of *S. cerevisiae* Dcp2, which spanned the Nudix motif, indeed catalyzed decapping *in vitro* (van Dijk et al., 2002). The Parker lab later echoed this finding and showed that recombinant Dcp1 enhances decapping by Dcp2 *in vitro* (Steiger et al., 2003). Together, these lead to the robust conclusion that Dcp2 is the catalytic subunit of the decapping enzyme, and Dcp1 is an important decapping co-factor. Immunostaining in human cells further revealed that Dcp1 and Dcp2 co-localize in distinct cytoplasmic puncta (van Dijk et al., 2002) later referred to as the Processing (P) bodies (Sheth and Parker, 2003). The physiological importance of decapping in eukaryotes was clearly reflected across species; characterized Dcp2 mutations are lethal in *S. cerevisiae*, *D. melanogaster* (herein: *Drosophila), D. rerio* and mice (Mishima and Tomari, 2017; Kim and van Hoof, 2020; Lee et al., 2020; Li et al., 2020), while alleles intriguingly lead to premature aging phenotypes in *C. elegans* (Rousakis et al., 2014; Li et al., 2020).

Not surprisingly, Dcp2 activity is heavily regulated by additional decapping factors. Several decapping activators were identified through genetic and proteomic studies, the first of which was PatL1 (Pat1) (Hatfield et al., 1996), and later followed by Edc1 and Edc2 (Dunckley et al., 2001), Edc3 (Kshirsagar and Parker, 2004), Ddx6 (Dhh1) (Fischer and Weis, 2002), Edc4 (Fenger-Gron et al., 2005), 4E-T (Dostie et al., 2000; Ferraiuolo et al., 2005), Pby1 (Sweet et al., 2007; Charenton et al., 2020) and Lsm14 (Scd6) (Decourty et al., 2008). Over the last 2 decades, biochemical interactions among the different decapping machinery members have been extensively studied across species and cell types. Notwithstanding the importance of these studies, much of the mechanistic insight was provided through structural studies on the decapping complex in yeast (*S. cerevisiae, K. lactis* and *S. pombe)* (She et al., 2008; Floor et al., 2010; Floor et al., 2012; Charenton et al., 2016; Mugridge et al., 2016; Valkov et al., 2016; Wurm et al., 2017; Mugridge et al., 2018b)*.*

## The Dcp1/2 Complex is the Main Decapping Holo-Enzyme in Eukaryotes

Eukaryotic mRNA 5′-caps can be subjected to the activities of several decapping enzymes belonging to four major families: 1) Nudix hydrolases, 2) Histidine Triad proteins, 3) DXO (Decapping and exoribonuclease) proteins and 4) ApaH-like phosphatases (reviewed in Kramer and McLennan, 2019). Histidine Triad decapping proteins include the abovementioned DcpS as well as FHIT, which together process the cap remnants that result from the 5′-3′ and 3′-5′ decay pathways (Taverniti and Seraphin, 2015). The DXO family proteins include the yeast Rai1 and Dxo1 proteins (Jiao et al., 2010; Chang et al., 2012), and a single known mammalian DXO ortholog (Jiao et al., 2013). These proteins perform an important cap quality control mechanism by selectively targeting incompletely capped mRNAs (Jiao et al., 2010; Chang et al., 2012; Jiao et al., 2013), and also process NAD^+^, FAD^+^ and dpCoA-capped mRNAs (Jiao et al., 2017; Doamekpor et al., 2020). The ApaH-like phosphatase is the major decapping enzyme that degrades the unique Cap 4 in *Trypanosoma* (Kramer, 2017), and does not seem to be functionally conserved outside of kinetoplastids (Castaneda Londono et al., 2021).

Dcp2 is a member of the Nudix hydrolase family, characterized by a loop-helix-loop Nudix hydrolase domain bearing the consensus motif GX_5_EX_7_REUXEEXGU, where U = bulky aliphatic residues and X = any amino acid (Bessman et al., 1996; Mildvan et al., 2005). This motif was initially characterized based on sequence alignment with the catalytic domain of the *E. coli* MutT protein, although most Nudix/MutT-domain-containing proteins have distinctive substrate specificities (Bessman et al., 1996). Among the 22 putative Nudix-domain-containing proteins in mammals, at least six can hydrolyze m^7^G cap *in vitro* (Song et al., 2013). In addition to Dcp2, only two others (Nudt3 and Nudt16) were validated as active *in vivo* (Song et al., 2010; Grudzien-Nogalska et al., 2016). A systematic comparison of global contributions of Dcp1/2, Nudt3 and Nudt16 has yet to be carried out in the same cell type and under the same conditions, but independent studies already suggest a broad transcriptome footprint for Dcp2, and a more selective impact for Nudt3 or Nudt16. For instance, a TimeLapse-seq study suggested that 1,803 transcripts are upregulated in HEK293 cells upon partial Dcp2 depletion (Luo et al., 2020). In contrast, an RNA-seq study identified 144 transcripts that were significantly upregulated upon Nudt3 knockdown in MCF-7 cell line (Grudzien-Nogalska et al., 2016), and microarray analyses on mouse embryonic fibroblast depleted for Nudt16 revealed the stabilization of 174 transcripts (Song et al., 2010). What underlies the target specificity and selectivity of each of these decapping enzymes is currently unknown.

### The Dcp2 Catalytic Core

**FIGURE 1:**
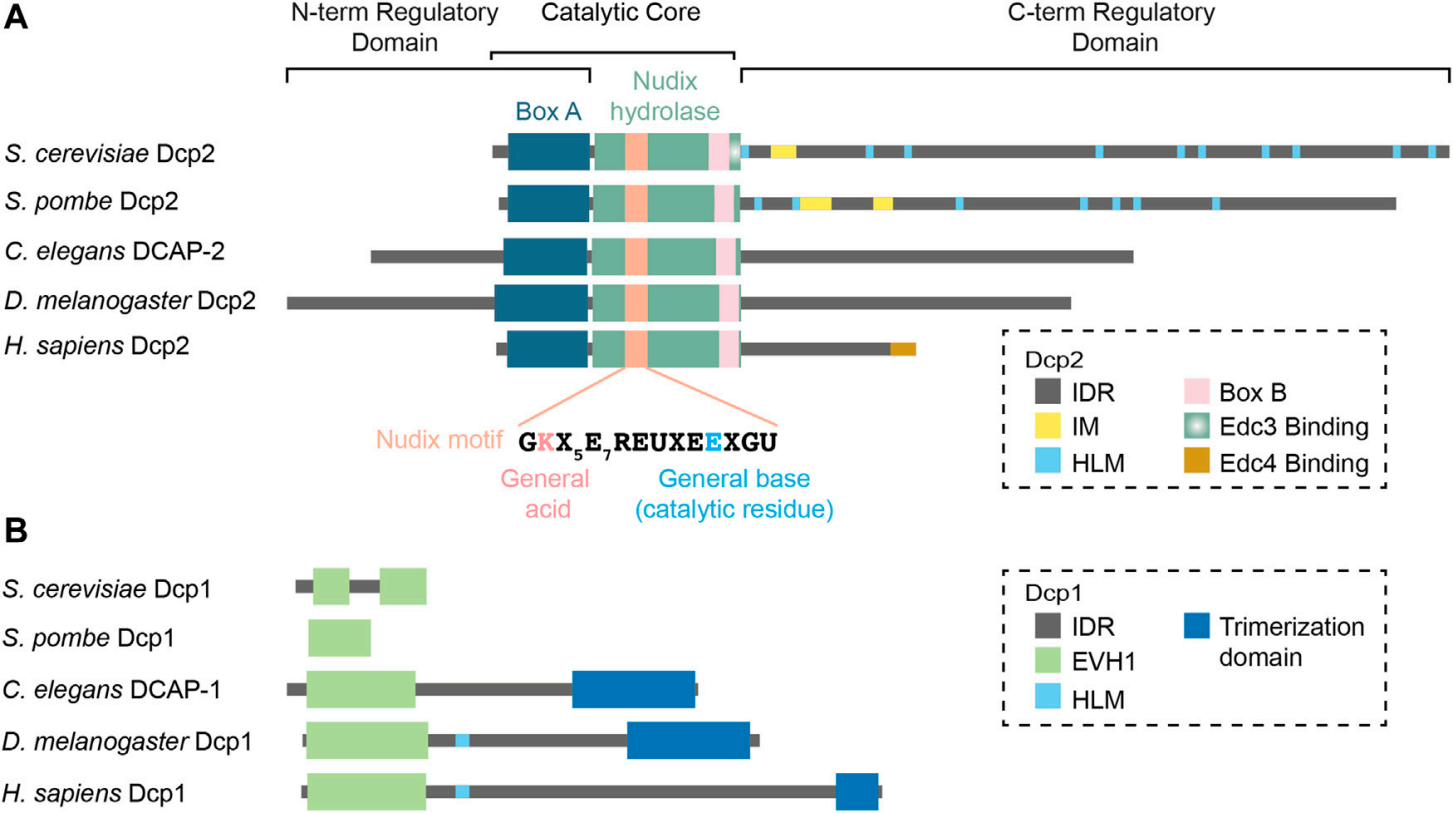
Domain organization of the decapping holocomplex in various organisms. The decapping holocomplex is composed of Dcp2 and Dcp1. **(A)** Dcp2 is the catalytic subunit that contains the catalytic core composed of the Nudix hydrolase domain and Box A. The N- and C-terminal ends are regulatory domains mainly composed of intrinsically disordered regions which vary in length and sequence in different species. **(B)** Dcp1 is the main activator of Dcp2 and is characterized by a conserved EVH1 domain. Metazoan Dcp1 acquires an intrinsically disordered extension in the C-terminus and can form a trimer. Abbreviations: IDR = Intrinsically Disordered Region; IM = Inhibitory Motif; HLM = Helical Leucine-rich Motif; EVH1 = Enabled/Vasodilator-stimulated Phosphoprotein Homology 1.

Dcp2 is thoroughly conserved in eukaryotes and remains the best studied eukaryotic decapping enzyme that is active preferentially on long m^7^G capped RNA substrates (van Dijk et al., 2002; Piccirillo et al., 2003; Cohen et al., 2004) (Figure 1A). It is also active *in vitro* on TMG-capped mRNAs, albeit with lesser efficiency than on m^7^G-capped mRNAs (Cohen et al., 2004). Dcp2 specifically cleaves the alpha-beta pyrophosphate bond of capped mRNAs, yielding m^7^GDP and 5′-monophosphorylated RNA (Lykke-Andersen, 2002; van Dijk et al., 2002; Wang et al., 2002). An invariant glutamic acid (*S. cerevisiae* E153, *S. pombe* E147) in the Nudix motif serves as a general base for catalysis and its mutation is sufficient to fully impair decapping activity *in vitro* and *in vivo* (Dunckley and Parker, 1999; Aglietti et al., 2013). An invariant lysine (*S. cerevisiae* K135, *S. pombe* K129) that functions as a general acid stabilizes the leaving group and is critical for RNA binding (Aglietti et al., 2013). The functional assignment of these residues is supported in the structure of the product (m^7^GDP)-bound Dcp2 where both residues are positioned near the beta-phosphate group of m^7^GDP (Charenton et al., 2016; Wurm et al., 2017). In the tertiary structure, four additional glutamic acid residues of the Nudix domain coordinate divalent cations (Mg^2+^ or Mn^2+^) that are required for catalysis (Aglietti et al., 2013). The Nudix motif is followed in C-terminus by a linker and a partially conserved Box B motif that is enriched in positively charged residues which forms an RNA-binding channel along with residues on the Nudix hydrolase domain (Wang et al., 2002; Deshmukh et al., 2008). In *S. cerevisiae*, the Box B motif is directly followed by a stretch of residues that is rich in hydrophobic amino acids or with long aliphatic side chains, which interact with Edc3 (Harigaya et al., 2010; Charenton et al., 2016). Meanwhile in *S. pombe*, binding to Edc3 mainly involves a region slightly downstream within the C-terminal intrinsically disordered region (IDR) (Fromm et al., 2012; Charenton et al., 2016), discussed below.

In Dcp2, the Nudix domain is preceded by a short and flexible hinge connected to a structured region often called the N-terminal Regulatory Domain (She et al., 2008; Floor et al., 2010; Charenton et al., 2016; Wurm et al., 2017; Mugridge et al., 2018b). This domain contains a highly conserved stretch of residues identified as Box A (Wang et al., 2002). Box A ensures the specificity of Dcp2 and its removal leads to aberrant production of m^7^GMP in addition to m^7^GDP *in vitro* (Piccirillo et al., 2003). Most importantly, the N-terminal regulatory domain harbors a set of conserved residues that interact with the cap which together with the Nudix hydrolase domain, form the composite active site of Dcp2 (Floor et al., 2010; Charenton et al., 2016; Wurm et al., 2017; Mugridge et al., 2018b). Keys to Dcp2 activity are an invariant “gatekeeper” tryptophan (*S. pombe* W43, *S. cerevisiae* W50) and a glutamic acid (*S. pombe* D47, *S. cerevisiae* D54) that are positioned on top of a loop called the 190s loop of the Nudix domain (Floor et al., 2010; Aglietti et al., 2013; Charenton et al., 2016; Wurm et al., 2017; Mugridge et al., 2018b). Together, these residues sandwich the cap when Dcp2 adopts an active conformation (Floor et al., 2010; Aglietti et al., 2013; Charenton et al., 2016; Wurm et al., 2017; Mugridge et al., 2018b). Other conserved residues are also located in the vicinity and interact with the phosphate groups of the cap (Charenton et al., 2016). Without a substrate, the cap-binding tryptophan (*S. pombe* W43) is blocked by a base-stacking interaction with an aromatic residue in the Nudix domain (*S. pombe* Y220, *S. cerevisiae* Y222) (Mugridge et al., 2016). This interaction is liberated upon substrate interaction, enabling both residues to engage with the cap (Mugridge et al., 2016). This aromatic residue also recognizes the first nucleotide of the capped mRNAs with a preference towards purines over pyrimidines (Mugridge et al., 2018b). In addition to containing residues that form the active site, the N-terminal regulatory domain of Dcp2 also interacts with Dcp1 via hydrophobic interactions (She et al., 2008). Interestingly, the N-terminal part of *Drosophila* and *C. elegans* Dcp2 also feature long intrinsically disordered regions (IDRs) (Figure 1A) but their significance for decapping catalysis and regulation is currently unknown.

### The Dcp2 Regulatory C-Terminal Intrinsically Disordered Region

The presence of a disordered C-terminal IDR is a common feature of eukaryotic Dcp2 proteins. The functions of the C-terminal IDR have been most extensively studied in yeast, where it serves both positive and negative regulatory functions through distinct elements. The positive regulatory elements are encoded as leucine-rich sequences commonly referred to as Helical Leucine-rich Motifs or HLMs (Gaudon et al., 1999). In *S. cerevisiae*, at least 10 HLMs were identified, first defined as short sequences bearing a core LLXΦL motif where Φ denotes any hydrophobic residue (Gaudon et al., 1999). *In vitro* pull-down assays demonstrated that each of these HLMs, except for HLMs 1 and 9, can directly interact with the decapping activator PatL1 (Pat1) (Charenton et al., 2017). *In vivo* deletions further suggested that HLMs 2–6 (equivalent to HLMs 1-5 in the He et al. study) contributed the bulk of PatL1 (Pat1) binding (He et al., 2021). Intriguingly, a strain bearing the deletion of HLMs 2–9 (equivalent to all HLMs in the He et al. study) still decays endogenous decapping targets as efficiently as wild-type (He et al., 2021). This could possibly be explained by a functional compensation by HLM1 and the Edc3 binding motif. In *S. pombe*, 7 putative HLMs have been identified (Fromm et al., 2012; Fromm et al., 2014). *S. pombe* HLMs can promote decapping activity *in vitro* by recruiting several decapping activators such as PatL1 and Edc3 with different affinities (Fromm et al., 2014; Lobel et al., 2019). Curiously, and unlike in *S. cerevisiae* and *S. pombe*, the C-terminal IDR of *Drosophila*, *C. elegans* and human Dcp2 lack any obvious HLMs. Human Dcp2 instead encodes a structured motif at the extreme C-terminal region, which recruits the decapping activator Edc4 (Chang et al., 2014). The rapid divergence of IDR and HLM sequences in Dcp2 C-terminus across species raises important questions on their possible functional redundancy or compensation by decapping activators.

In addition to HLMs, the C-terminal IDR of *S. cerevisiae* and *S. pombe* Dcp2 also contains negative *cis* regulatory elements. An inhibitory element, often referred to as Inhibitory Motif, was first reported by the Jacobson lab in a complementation experiment revealing that certain truncated fragments of Dcp2 were constitutively active and could bypass the requirement for Edc3 (He and Jacobson, 2015). Systematic deletions of the C-terminal IDR further mapped the inhibitory motif to a 25-amino acid region enriched in prolines and phenylalanines (He and Jacobson, 2015). More recently, the Gross lab identified two inhibitory motifs in *S. pombe* Dcp2, one similar to the *S. cerevisiae* inhibitory motif and the other that is exclusively found in closely related *Schizosaccharomyces* species (Paquette et al., 2018). Their structural implications remain unclear due to lack of crystal structures of these motifs in the context of the full-length active or inactive Dcp2. Nonetheless, at least one of the inhibitory motifs in *S. pombe* could interact with a Dcp2 fragment containing the N-terminal regulatory and the Nudix hydrolase domains (Paquette et al., 2018), supporting a possibility that they may block the active site.

Whether metazoan Dcp2 harbors such inhibitory motifs remains to be confirmed. However, the C-terminal 60 amino acids of human Dcp2 are subjected to ubiquitination and subsequent proteasomal degradation of Dcp2 (Erickson et al., 2015). Thus, across a wide range of species, Dcp2 is subjected to both positive and negative regulation by its C-terminus through diverse, and possibly diverging, mechanisms.

### Dcp1

Dcp1 is the regulatory subunit of the Dcp1/2 holoenzyme (She et al., 2008). In *S. cerevisiae* and *S. pombe*, Dcp1 is essential for decapping activation and strains wherein Dcp1 is deleted exhibit severely impaired decapping activity (Beelman et al., 1996; Sakuno et al., 2004). Dcp1 interacts directly with Dcp2 in *S. cerevisiae* and *S. pombe* (She et al., 2008) and strongly potentiates Dcp2 activity *in vitro* (Steiger et al., 2003). In contrast, human Dcp1 only interacts with Dcp2 with low affinity, but their interaction is stabilized by Edc4 (Chang et al., 2014). This three-way interaction is required for decapping *in vitro* and mutations that specifically impair the interface between Dcp1 and Dcp2 fails to rescue the degradation of a reporter mRNA (Chang et al., 2014). The requirement for Edc4 likely explains earlier observations that recombinant Dcp1 from *C. elegans*, *Drosophila* and human cells are insufficient to promote catalysis by Dcp2 *in vitro* (van Dijk et al., 2002; Cohen et al., 2004; Lin et al., 2008). Thus, Dcp1 promotes Dcp2 activity in yeast and metazoans, but their functional architecture seems to diverge and involve additional cofactors in metazoans (Chang et al., 2014).

Dcp1 is primarily characterized by an EVH1 domain (Callebaut, 2002) that mediates the interaction with the N-terminal regulatory domain of Dcp2 (She et al., 2004; She et al., 2008) (Figure 1B). In *Drosophila* and possibly in other species, the EVH1 domain of Dcp1 also interacts with proline-rich sequences in the exonuclease Xrn1, thus physically coupling decapping and decay (Braun et al., 2012). In *S. cerevisiae* and *S. pombe*, Dcp1 is solely composed of the EVH1 domain (Callebaut, 2002). Interestingly, metazoan Dcp1 proteins also contain a longer IDR extension, a difference that is mirrored by the shortening of the Dcp2 C-terminal IDR. Furthermore, the IDR of Dcp1 in *Drosophila* and humans encodes an HLM that can interact with Edc3, further suggesting that some of the functions of Dcp2 IDR have been transferred over to Dcp1 in metazoans (Tritschler et al., 2009a; Fromm et al., 2012). The extreme C-terminus of metazoan Dcp1 further encodes a structured region that enables trimerization and is required for Dcp2 to interact with Edc4 (Tritschler et al., 2009b). However, the molecular basis for the interface between metazoan Dcp2 and the trimeric form of Dcp1 remains to be determined structurally.

## Decapping Activators

**FIGURE 2:**
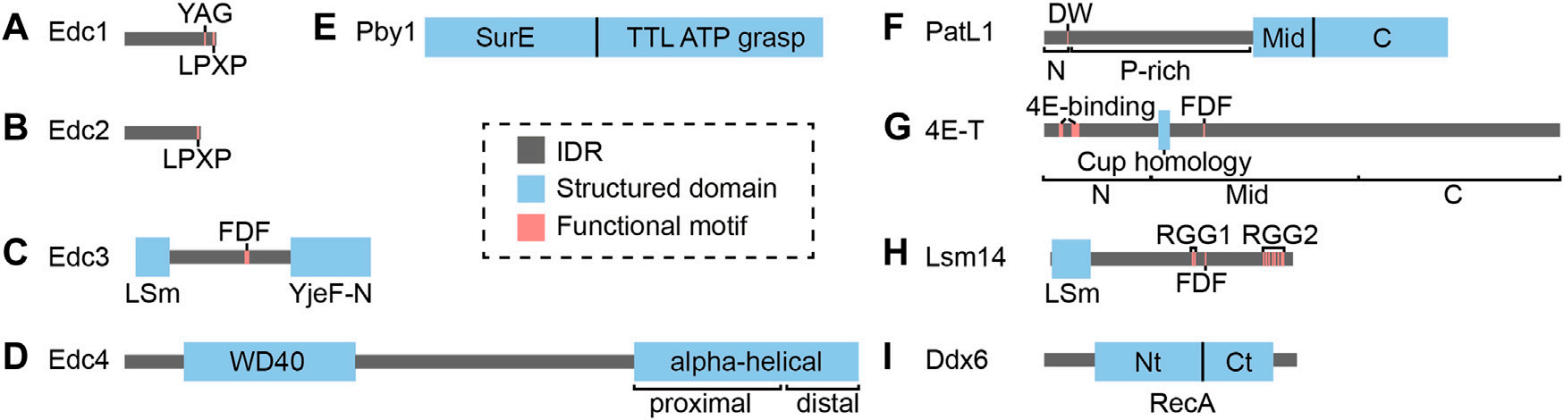
Domain organization of various decapping activators. The archetypal domain organization of **(A)** Edc1, **(B)** Edc2, **(C)** Edc3, **(D)** Edc4, **(E)** Pby1, **(F)** PatL1, **(G)** 4E-T, **(H)** Lsm14 and **(I)** Ddx6 are illustrated. Depicted are the *S. cerevisiae* orthologs of Edc1, Edc2 and Pby1, and the *H. sapiens* orthologs of the other decapping activators. Conserved domains and motifs are highlighted, and species-specific features are discussed in the main text. Abbreviation: IDR = Intrinsically Disordered Region.

**FIGURE 3:**
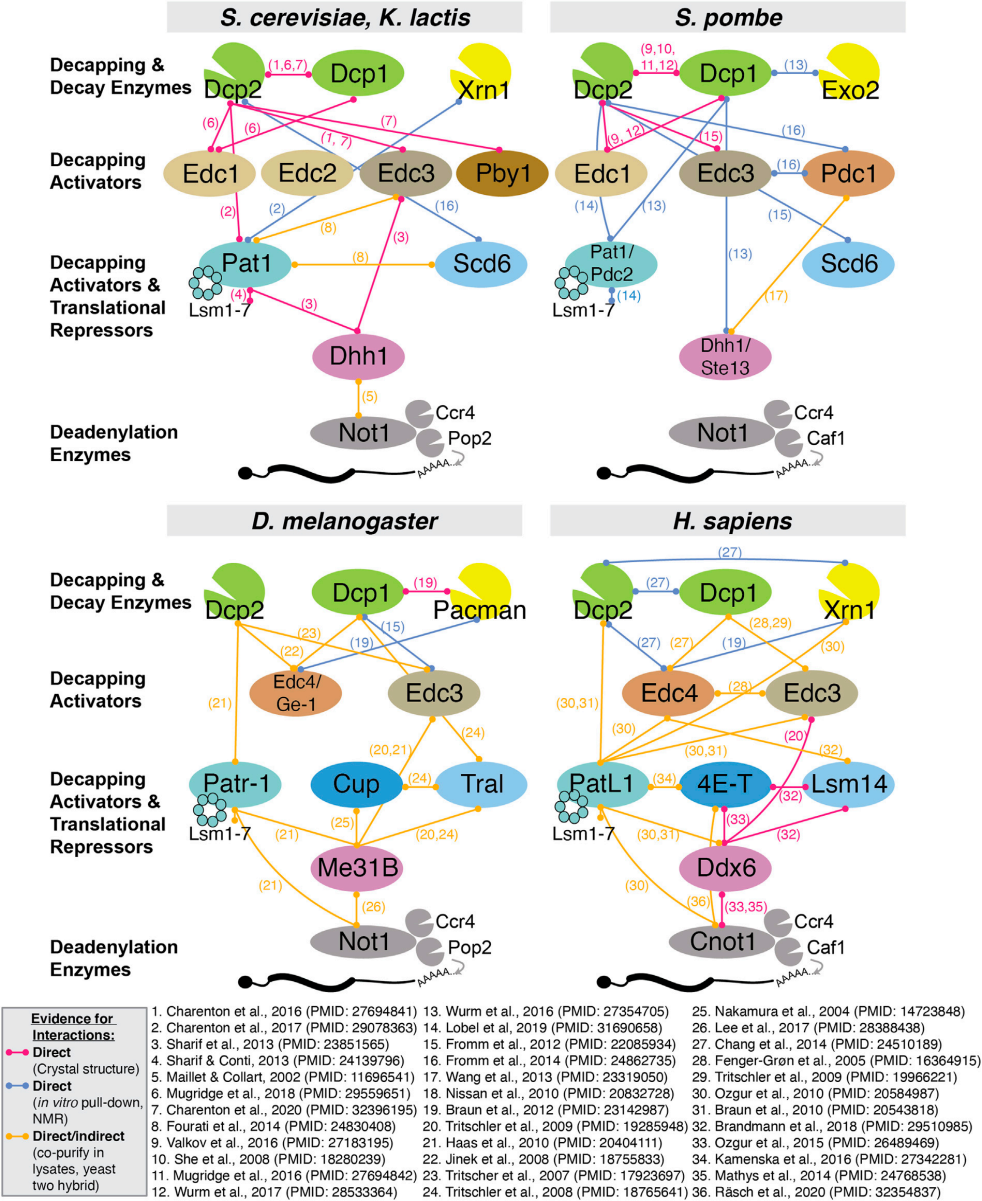
Summary of all reported interactions between decapping proteins in *S. cerevisiae*, *S. pombe*, *D. melanogaster* and *H. sapiens*. Due to space constraints, we only include evidence from experimentally validated interactions and indicate whether evidence for direct physical interaction has been reported.

An over-arching theme in mRNA decapping mechanisms is the recruitment and potentiation of the Dcp1/2 decapping holoenzyme by activating proteins that often also serve as scaffolds on silenced mRNAs (Figure 2). While the Edc1-4 and Pby1 activators only seem to enhance decapping, others such as PatL1, 4E-T, Lsm14 and Ddx6 instead appear to have dual functions, as they can also protect mRNAs from degradation and keep them in a translationally repressed state (He and Parker, 2001; Coller and Parker, 2005; Tanaka et al., 2006; Igreja and Izaurralde, 2011; Kamenska et al., 2014; Wang et al., 2017; Räsch et al., 2020). Part of this apparent paradox can be explained by the ability of this set of decapping activators to not only interact with Dcp1/2 or Edc1-4 proteins, but also with translational repression factors such as cap-binding proteins (Dostie et al., 2000; Kubacka et al., 2013). The fate of their target mRNAs is not only determined by the recruitment of specific decapping enhancers to the Dcp1/2 enzyme but also by the availability or affinity of interacting partners, which may vary with cellular and developmental contexts. Here, we discuss the interactions and functions of decapping activators, highlighting the similarities and differences across cell types and species. Figure 3 summarizes interactions of decapping activators, contrasts species-specific distinctions, and points to key missing links.

### Edc1 and Edc2

Edc1 and Edc2 are encoded by loci that were identified as suppressors of Dcp1/2 mutations in *S. cerevisiae* (Dunckley et al., 2001). They encode small, intrinsically disordered proteins sharing 42% amino acid similarity (Dunckley et al., 2001), that enhance decapping *in vitro* by 140- and 40-fold, respectively (Borja et al., 2011), and can bind RNA (Schwartz et al., 2003). The decapping enhancement function of Edc1 was attributed to two of its motifs, namely an LPXP motif which interacts with Dcp1 EVH1 domain (Borja et al., 2011; Valkov et al., 2016; Wurm et al., 2016), and a YAG activation motif that binds in a groove between the Dcp2 N-terminal regulatory and Nudix hydrolase domains, thereby stabilizing the cap-binding pocket (Wurm et al., 2017; Mugridge et al., 2018b) (Figure 2A). The latter contributes to an increase in the affinity of the Dcp1/2 holocomplex towards RNA, which in turn enhances catalysis (Wurm et al., 2016; Wurm et al., 2017). In contrast, *S. cerevisiae* Edc2 lacks the YAG motif and how it promotes decapping is not well understood (Figure 2B). Both mammalian homologs of Edc1/2, PNRC1 and PNRC2, contain the LPXP and YAG motifs and are likely to activate Dcp1/2 via similar mechanisms. Curiously, PNRC1 and PNRC2 seem to have specialized in distinct RNA decapping functions. On one hand, human PNRC1 is predominantly nuclear and can recruit Dcp1/2 complex to the nucleolus, where this interaction was proposed to effect decapping of the U3 and U8 snoRNAs (Gaviraghi et al., 2018). On the other hand, PNRC2 is cytoplasmic and localizes to P bodies (Cho et al., 2009), but it forms a complex and functions with a different set of decapping activators, including Upf1 and Smg5, which are involved in the nonsense-mediated decay (NMD) pathway (Lai et al., 2012; Cho et al., 2013). Any link between PNRC1 and PNRC2 with other decapping activators, and whether they are involved more generally in mRNA decapping pathways outside of NMD, remains to be determined.

### Edc3

Edc3 was implicated in decapping through several independent large-scale proteomic surveys in *S. cerevisiae,* which detected interactions with decapping proteins Dcp1, Dcp2, PatL1 (Pat1) and the Lsm proteins (Fromont-Racine et al., 2000; Uetz et al., 2000; Gavin et al., 2002). Its function in decapping activation was first demonstrated in *S. cerevisiae* where deletion of Edc3 exacerbates the decapping impairment caused by hypomorphic Dcp1 or Dcp2 alleles (Kshirsagar and Parker, 2004), and recombinant Edc3 clearly potentiates decapping by Dcp1/2 complex *in vitro* (Harigaya et al., 2010; Nissan et al., 2010; Fromm et al., 2012). In human cells, Edc3 also localizes to P bodies and promotes decapping by Dcp2 (Fenger-Gron et al., 2005).

Edc3 (Figure 2C) encodes an N-terminal LSm (Sm-like) domain which, unlike the canonical Sm motif, neither multimerizes into Sm rings nor binds RNA, but rather accommodates different protein-protein interactions (Tritschler et al., 2007). This domain interacts directly with a region immediately upstream of the first HLM of Dcp2 in *K. lactis* and *S. cerevisiae* (Charenton et al., 2016), with the HLMs themselves in *S. pombe*, or with the Dcp1 HLM in metazoans (Fromm et al., 2012; Fromm et al., 2014). Downstream of the LSm domain of Edc3 is a long IDR that harbors a conserved FDF (phenylalanine-aspartic acid-phenylalanine) motif and interacts with Ddx6 (Tritschler et al., 2009a). Lastly, the C-terminal end of Edc3 is characterized by a YjeF-N domain with a Rossman fold topology that facilitates Edc3 homodimerization (Ling et al., 2008) and in turn promotes phase separation *in vitro* (Tibble et al., 2021). *In vivo*, Edc3 appears to act redundantly with another LSm domain-containing decapping activator, Lsm14 (*S. cerevisiae* Scd6, detailed below), as decapping is only impaired when both proteins are deleted simultaneously (Decourty et al., 2008).

Interestingly, *S. cerevisiae* Edc3 can promote the degradation of two specific transcripts in an atypical and deadenylation-independent manner. Firstly, a short motif on Edc3 that is conserved among Saccharomycetaceae species can interact with the ribosomal protein Rps28, and together bind to a hairpin in the 3′-untranslated region of the *rps28b* mRNA to promote its degradation (Badis et al., 2004; Kolesnikova et al., 2013; He et al., 2014). This Edc3-mediated degradation of *rps28b* requires translation and thus presents intriguing autoregulation of ribosomal protein levels that directly involves decapping cofactors (He et al., 2014). Secondly, Edc3 promotes the degradation of nuclear export factor *YRA1* pre-mRNA, which uniquely evades NMD in the cytoplasm (Dong et al., 2007). The mechanism likely involves direct recruitment of Edc3 to *cis* elements in the *YRA1* intron following translational repression (Dong et al., 2010).

### Edc4

Edc4 was identified as a component of P bodies in human cells, where it co-localizes with Dcp1/2 (Fenger-Gron et al., 2005; Yu et al., 2005). It was also captured in a screen for miRNA-mediated silencing components in *Drosophila* S2 cells (Eulalio et al., 2007b). Edc4 (Figure 2D) encodes an N-terminal WD40 domain that arranges as a circularized seven-bladed beta-propeller and facilitates interaction with Dcp1 trimers (Chang et al., 2014). This domain is successively followed by a serine-rich linker and a C-terminal alpha-helical hairpin repeat similar to those found in ARM and HEAT-repeat proteins (Jinek et al., 2008). In humans, the proximal C-terminus of Edc4 promotes Edc4 oligomerization, as well as interacts directly and simultaneously with Dcp2 and Xrn1 (Chang et al., 2014). These interactions are mediated by short Edc4-binding motifs in Dcp2 and Xrn1 (Braun et al., 2012; Chang et al., 2014). Interestingly, the Edc4-binding motif in Dcp2 is not conserved in *Drosophila* and *C. elegans* (Chang et al., 2014), and thus how Edc4 interacts with Dcp2 in these organisms remain unclear. In humans, Edc4 interaction with Xrn1 also alleviates the inhibition of deadenylation caused by Xrn1-mediated sequestration of Caf1, one of the catalytic deadenylation subunit (Chang et al., 2019). Therefore, Edc4 could indirectly enhance decapping by promoting deadenylation, and more directly by scaffolding decapping and decay enzymes. Recently, Edc4 was involved in the inhibition of a novel mRNA decay pathway initiated by the endonuclease MARF1 (Brothers et al., 2020). Edc4 can thus serve both as an enhancer of decapping and as a repressor of mRNA decay pathways.

Edc4 does not have any clear ortholog in *S. cerevisiae*, but the *S. pombe* Pdc1 protein exhibits commonalities with Edc4 (Wang et al., 2013; Fromm et al., 2014). Pdc1 bears a WD40-repeat and a distal C-terminus that folds into helical repeats found in *Drosophila* Edc4 despite sharing only 17% of sequence identity (Fromm et al., 2014). Pdc1 also interacts with the LSm domain of Edc3 through at least three HLMs encoded in its N-terminus (Fromm et al., 2014), and co-localizes with other decapping factors to P bodies (Wang et al., 2013). Whether and how Pdc1 directly activates the Dcp1/2 holoenzyme in *S. pombe* remains to be determined. A structural comparison of Edc4 and Pdc1, alone or in interaction with Dcp1/2, could likely prove insightful.

### Pby1

Pby1 is a decapping activator in yeast that is related to the human tubulin tyrosine ligase (TTL) protein (Sweet et al., 2007; Rao and Parker, 2017; Charenton et al., 2020). Pby1 involvement in mRNA decapping and decay regulation was suggested by its association with P bodies through large-scale interactome studies (Gavin et al., 2006; Decourty et al., 2008). This was confirmed by fluorescence imaging of *S. cerevisiae* where Pby1 co-localizes with Dcp2 in P bodies (Sweet et al., 2007). Pby1 is composed of an N-terminal domain that resembles the SurE phosphatase family (Iwasaki and Miki, 2007) and a C-terminal domain consisting of an ATP-grasp fold (Fawaz et al., 2011) (Figure 2E). Interestingly, the structure of the Pby1-Dcp1-Dcp2-Edc3 complex of *S. cerevisiae* revealed that several conserved residues in the Pby1 C-terminal domain interact with the Nudix domain of Dcp2 (Charenton et al., 2020). Moreover, point mutations that impair this interaction disperse Pby1 from P bodies to the cytoplasm (Charenton et al., 2020). *In vivo*, the decapping-promoting function of Pby1 is inferred from the observation that Pby1 overexpression restores the growth defect phenotype of decapping mutants in *S. cerevisiae*, namely a double deletion of PatL1 and Ddx6 (Dhh1) and a triple deletion of PatL1, Edc3 and Lsm14 (Scd6) (Charenton et al., 2020). This effect is critically contingent upon Pby1 interaction with Dcp2 (Charenton et al., 2020).

How Pby1 precisely impacts Dcp2 activity remains an important question to address. The catalytic activity of the ATP-grasp domain is dispensable for function *in vivo*, and *in vitro* decapping assays did not show a direct enhancement in decapping activity upon addition of recombinant Pby1, possibly due to a missing co-factor (Charenton et al., 2020). Furthermore, Pby1 deletion did not significantly alter the stability of reporter mRNAs (Sweet et al., 2007). One possibility could be that Pby1 functions predominantly in certain cellular states or when other decapping activators are involved, as suggested by its genetic interactions (Charenton et al., 2020). The latter is also in line with the observation that Pby1 could drive Dcp2 localization into P bodies in the absence of Edc3 and Lsm14 in *S. cerevisiae* (Rao and Parker, 2017). Assaying the decapping-promoting function of Pby1 in mutant backgrounds might provide further mechanistic and regulatory insights. Lastly, whether metazoan orthologs of Pby1 are involved in decapping, or whether other metazoan proteins might serve functions that are orthologous to Pby1 would be interesting avenues of investigation.

### PatL1 (Pat1) and Lsm1-7

PatL1 (Pat1) was linked to decapping through a genetic screen for *S. cerevisiae* mutants that fail to degrade an unstable *MFA2pG* reporter mRNA (Hatfield et al., 1996), and independently in a suppressor screen that renders a Pab1 deletion viable (Boeck et al., 1998; Bonnerot et al., 2000). Lsm1, which is part of the cytoplasmic Lsm1-7 complex (Sharif and Conti, 2013; Wu et al., 2014), was also captured in the latter screen, and as with PatL1 and Dcp2, its deletion strongly inhibits decapping (Bouveret et al., 2000; Tharun et al., 2000). Furthermore, strains bearing deletions of Lsm1 or PatL1 have very similar transcriptome profiles (He et al., 2018). The decapping and decay role for metazoan PatL1 was robustly established by the observation that tethering of PatL1 leads to destabilization of reporter mRNAs both in *Drosophila* and human cells (Haas et al., 2010; Ozgur et al., 2010).

Structurally, the PatL1 protein can be divided into four functional regions: N-terminal, proline-rich, Mid and C-terminal domains (Haas et al., 2010) (Figure 2F). The N-terminal domain is a predicted disordered region and harbors an FDF motif (equivalent to the DW motif in metazoan PatL1) that binds directly to Ddx6 (Dhh1) (Haas et al., 2010; Sharif et al., 2013). This domain is dispensable for decapping activity and for *S. cerevisiae* growth (Pilkington and Parker, 2008), likely due to redundancy of decapping mechanisms that converge on Ddx6 (Sharif et al., 2013). The proline-rich region of PatL1 possibly interacts with the EVH1 domain of Dcp1 (Braun et al., 2012), which may provide a direct link to the decapping holoenzyme. The Mid and C-terminal domains are conserved across species and fold into a helical organization (Braun et al., 2010; Fourati et al., 2014; Wu et al., 2014). The Mid domain co-immunoprecipitates with the CCR4-NOT deadenylase complex (Haas et al., 2010), but whether this involves direct binding remains to be determined. A highly conserved motif in the N-terminal portion of the C domain, along with some residues in the Mid domain, provides a bipartite module for binding to the Lsm1-7 complex (Braun et al., 2010; Fourati et al., 2014). This interaction promotes decapping by stabilizing the binding of Lsm1-7 to RNA (Chowdhury et al., 2007; Lobel et al., 2019). In yeast, the other end (C-terminal) of the PatL1 C-terminal domain consists of a yeast-specific motif that interacts directly with the HLMs of Dcp2 and Xrn1 (Charenton et al., 2017; Lobel et al., 2019). This interaction mirrors the function of metazoan Edc4 (Chang et al., 2014) in coupling decapping with exonucleolytic degradation. PatL1 interaction with Dcp2 was proposed to alleviate decapping autoinhibition, possibly by altering the conformation of the C-terminal IDR of Dcp2 (Charenton et al., 2017; Lobel et al., 2019). Point mutations that impair the HLM-binding sites on PatL1 significantly impair decapping *in vitro* and *in vivo* (Charenton et al., 2017). Together, the Mid and C-terminal domains are sufficient for PatL1 function *in vivo* (Pilkington and Parker, 2008).

In addition to its decapping and decay scaffolding functions, the affinity and preference of the PatL1/Lsm1-7 complex for deadenylated RNAs provide a rationale for the functional linkage of the decapping machinery with deadenylated mRNAs. *In vitro*, a PatL1/Lsm1-7 octamer binds the 3’ end of mRNAs with greater affinity towards oligoadenylated (<10 terminal adenines) than polyadenylated mRNAs, and an even greater affinity towards RNA bearing a stretch of ∼6 uracils (Chowdhury et al., 2007). Although the Lsm1-7 complex seems to prefer U-rich sequences on its own, PatL1 broadens its affinity towards more A-rich sequences (Lobel et al., 2019; Lobel and Gross, 2020). This agrees with RNA-seq in human cells where PatL1 depletion preferentially stabilizes AU-rich mRNAs (Vindry et al., 2017).

In contrast to a role in decapping activation, a PatL1/Lsm1-7 complex has also been involved in protecting deadenylated mRNAs from degradation (He and Parker, 2001) and in keeping transcripts in a translationally repressed state in *S. cerevisiae* (Coller and Parker, 2005) and during early oocyte development in *X. laevis* (Marnef et al., 2010). Under hyperosmotic stress, the PatL1/Lsm1-7 complex is recruited to stress-induced transcripts to repress their translation without impacting mRNA stability, and deletion of PatL1 or Lsm1 reactivates the translation of these transcripts (Garre et al., 2018). Evidence supports a similar function in metazoans. In *Drosophila*, PatL1 associates with the Ddx6 (Me31B) and GIGYF proteins to repress translation (Peter et al., 2019). The same interaction between PatL1 (PATR-1) and GIGYF (GYF-1) has also been reported in *C. elegans*, where GYF-1 represses translation of some developmental miRNA targets (Mayya et al., 2021). Thus, promotion of decapping and decay or translational repression by PatL1 seems to depend on its specific interactions and on environmental and developmental contexts.

Lastly, the functions of PatL1 and Lsm1-7 proteins in mammals extend beyond their involvement in mRNA silencing. For example, while human PatL1 is predominantly (∼80%) cytoplasmic, the nuclear PatL1 pool associates with Lsm2-8, U6 snRNA and SART3 as part of the U6 snRNP in Cajal body (Vindry et al., 2017). This complex promotes exon cassette inclusion, with knockdown of PatL1 in cells leading to changes in about 180 alternative splicing events with weak splice donor sites (Vindry et al., 2017).

### 4E-T (Cup)

4E-T (Cup) is a metazoan-specific protein that has dual roles in translational repression and decapping & decay by virtue of its direct interactions with translational repressors and decapping co-factors. Most of its sequence is unstructured and poorly conserved, but 4E-T features two eIF4E-binding sites (a canonical YXXXXLϕ and a non-canonical 4E-binding motif) in the N-terminus (Dostie et al., 2000; Nelson et al., 2004), and a conserved 4E-T (Cup) Homology Domain (Kamenska et al., 2014) (Figure 2G). The two eIF4E-binding motifs of 4E-T can also interact with another cap-binding protein, the translational repressor 4EHP (Kubacka et al., 2013). Two separate motifs in the 4E-T Mid and C-terminal regions also interact with Ddx6 and Lsm14 (Nishimura et al., 2015), which in turn can interact with other decapping activators (Brandmann et al., 2018). The importance of 4E-T in mRNA decapping and decay is supported by the observation that knockdown of 4E-T stabilizes Tristetrapolin (TTP)- and miRNA-mediated decay reporter mRNAs in HeLa cells (Ferraiuolo et al., 2005; Nishimura et al., 2015). Mechanistically, this decapping and decay enhancement is thought to function through the competitive release of the 5′-cap from the translation initiation complex (eIF4E, 4G, 4A), rendering the cap accessible to decapping complex (Ferraiuolo et al., 2005; Nishimura et al., 2015).

In contrast with its role in decapping enhancement, substantial evidence indicates that 4E-T can also protect target mRNAs from decapping and decay. In *Drosophila, in situ* hybridization and qRT-PCR results indicated that targeted mRNAs are destabilized upon depletion of 4E-T (Cup), or upon mutation in its eIF4E-binding motif (Broyer et al., 2017). In *Drosophila* S2 cells, 4E-T (Cup)-bound mRNAs are deadenylated and capped, but are destabilized when the eIF4E-binding motif of 4E-T (Cup) is mutated (Igreja and Izaurralde, 2011). Curiously, transfection of a construct encoding only the Mid or C domain leads to deadenylation, decapping and decay of Cup-bound mRNAs, suggesting that the N-terminus of 4E-T inhibits decapping and decay (Igreja and Izaurralde, 2011). Similarly, in human HEK293 cells, tethering of 4E-T also resulted in the stabilization of reporter mRNAs bearing an AU-rich element or miRNA-binding sites (Räsch et al., 2020). This result stands at odds with earlier tethering experiments (Ferraiuolo et al., 2005; Nishimura et al., 2015). Mechanistically, 4E-T could repress mRNA translation by increasing the affinity of 4EHP towards the cap (Chapat et al., 2017), which in turn could block Dcp1/2 from accessing the cap.

A possible explanation for the alternative fates of 4E-T–bound mRNAs could lie in the availability and binding affinities of eIF4E and 4EHP to 4E-T. Perhaps in certain cell types or under specific conditions where 4EHP is abundant or more readily interacts with 4E-T, protection from decapping could be favored for 4E-T–bound mRNAs. It is also possible that this affinity for the cap can be modulated through conformational changes due to other interactions with 4E-T. Better structural insight on 4E-T and its interacting partners and precise quantification of the affinity of the 4E-T/4EHP complex in its native niche will likely be required to shed light on the role of 4E-T in mRNA translational repression, decapping and decay.

### Lsm14 (Rap55/Scd6/Trailer Hitch)

The Lsm14 (Rap55) protein was first identified as a component of mRNP complexes in oocytes and early embryos of the amphibians *Pleurodeles waltl* and *X. laevis* (Lieb et al., 1998), and later as an essential component of Dcp1 and Edc4-containing P bodies in human cells (Bloch et al., 2005; Tanaka et al., 2006; Yang et al., 2006). The *S. cerevisiae* ortholog of Lsm14 (Scd6) was implicated in decapping through a screen for growth defects under a null allele of Edc3 (Decourty et al., 2008).

Lsm14 (Figure 2H) is related to Edc3 in sharing an N-terminal LSm domain (Albrecht and Lengauer, 2004). In *S. cerevisiae* Lsm14 (Scd6), this domain is necessary and sufficient to promote the decapping of a reporter mRNA (Zeidan et al., 2018) and interacts with the HLMs of Dcp2 (Fromm et al., 2012). The orthologous domain interacts with the EVH1 domain of Dcp1 in metazoans (Tritschler et al., 2008). In contrast with Edc3, which ends with a structured YjeF-N dimerization domain, the C-terminal end of Lsm14 consists of a long IDR that harbors an FDF motif and RGG repeats that vary in numbers in different species (Marnef et al., 2009). Metazoan Lsm14 features two clusters of RGG repeats that sandwich the FDF motif, while the yeast ortholog (Scd6) has only one RGG repeat located downstream of the FDF motif (Lieb et al., 1998; Marnef et al., 2009). RGG motifs are important for Lsm14 to localize to P bodies, but their contribution seems to vary between cell types or experimental conditions (Tanaka et al., 2006; Yang et al., 2006; Matsumoto et al., 2012). For example, both RGG clusters are necessary and sufficient in human Hep-2 cell lines (Yang et al., 2006) but seem to have a lesser impact in HeLa cells (Tanaka et al., 2006; Matsumoto et al., 2012). Although the precise mechanism of Dcp1/2 enhancement by Lsm14 is currently poorly understood, Lsm14 (Scd6) and Edc3 in *S. cerevisiae* maintain a functional pool of Dcp2 in the cytoplasm, as their combined deletion results in nuclear retention of inactive Dcp2 (Tishinov and Spang, 2021).

In addition to decapping enhancement, Lsm14 also mediates translational repression when tethered to reporter mRNAs in *X. laevis* oocytes (Tanaka et al., 2006) and *S. cerevisiae* (Zeidan et al., 2018). In *S. cerevisiae*, this function is attributed to the interaction between the RGG motifs on Lsm14 with eIF4G within the eIF4F complex, thereby repressing the assembly of 48S initiation complex (Rajyaguru et al., 2012). In *Drosophila* and human Lsm14, the LSm domain recruits 4E-T (Cup), and a bipartite phenylalanine-rich motif (FDF and TFG) in the Lsm14 IDR segment interacts with Ddx6 (Me31B) (Tritschler et al., 2008; Brandmann et al., 2018). The three proteins (Lsm14, Ddx6 and 4E-T) form a highly conserved translational repressor complex (Tritschler et al., 2008; Brandmann et al., 2018). In *Drosophila* and *C. elegans* early embryonic development, this complex represses a subset of maternal mRNAs (Boag et al., 2008; Wang et al., 2017), and mutants of the *C. elegans* Lsm14 ortholog (CAR-1) die early in embryogenesis due to failed cytokinesis (Audhya et al., 2005).

Since Lsm14 utilizes the LSm domain to interact with both Dcp1 and 4E-T (Cup) (Tritschler et al., 2008), the capacity of Lsm14 to induce decapping or translational inhibition might be a consequence of the relative affinities and cellular availabilities of Dcp1/2, 4E-T and Ddx6. This hypothesis could be tested through detailed quantification of their expression in different cellular and developmental contexts.

### Ddx6 (Dhh1/Me31B)

Ddx6 (Dhh1/Me31B) is a DEAD-box helicase that functionally intersects with deadenylation, decapping activators, and translational repression across multiple eukaryotic species (Weston and Sommerville, 2006). Ddx6 (Figure 2I) and its orthologs are characterized by a core helicase composed of two RecA-like domains separated by a short linker (Ostareck et al., 2014). Like other DEAD box helicases, the Ddx6 active site is formed when the two RecA domains come into close interaction in the presence of ATP and RNA (Ostareck et al., 2014). Unlike other DEAD box helicases, Ddx6 has a weak ATPase activity and needs additional factors to be activated (Dutta et al., 2011). More specifically, direct binding of the MIF4G domain of CNOT1, a scaffold subunit of the CCR4-NOT deadenylase complex, to the C-terminal RecA domain of Ddx6 (Dhh1) changes the active site conformation and activates ATP hydrolysis (Maillet and Collart, 2002; Mathys et al., 2014; Mugler et al., 2016). Mutation of the CNOT1/Ddx6 binding interface de-represses a reporter mRNA bearing *let-7* miRNA binding sites; hence ATPase activity is important for silencing through Ddx6 (Dhh1) (Mathys et al., 2014; Rouya et al., 2014).

Ddx6 can promote decapping and decay through its extensive interactions with decapping activators. The C-terminal RecA domain of Ddx6 can bind to Edc3, PatL1, Lsm14 and 4E-T using overlapping binding interfaces (Tritschler et al., 2008; Sharif et al., 2013; Ozgur et al., 2015). The possibility of competitive binding to Ddx6 suggests that it may split the partner proteins into distinct complex populations, enacting either translational repression (in the case of PatL1, Lsm14, or 4E-T) or decapping (in the case of Edc3, PatL1, Lsm14, or 4E-T) on deadenylated mRNAs. Since ATP hydrolysis by DEAD box helicases leads to reduced RNA binding (Hondele et al., 2019), activation of the Ddx6 ATPase by CNOT1 (Not1) may release the deadenylated mRNA to be accessible for the recruitment of other Ddx6-associated decapping activators.

Interestingly, the interaction between Ddx6 and the CCR4-NOT scaffold subunit is thus far the only direct physical link between the deadenylation and decapping machineries. This is a critical concept, as mRNA deadenylation is a typical prelude to decapping and decay (Muhlrad et al., 1994; Garneau et al., 2007). Deadenylation is triggered by a wide variety of elements in the 3′-untranslated region and their associated factors (Mayya and Duchaine, 2019). The coupling of deadenylation, decapping and decay is seen in a variety of decay pathways including silencing through the miRNA Induced Silencing Complex, and by RNA-binding proteins such as Pumilio (Van Etten et al., 2012) and AU-rich associated Tristetrapolin (Sandler et al., 2011). These factors directly recruit the deadenylase complex through CNOT1 (Braun et al., 2011; Fabian et al., 2013; Enwerem et al., 2021), but among the RNA binding proteins above, only Tristetrapolin is known to directly interact with Dcp2 thus far (Maciej et al., 2021). Since Ddx6 is a highly abundant protein in cells (Ghaemmaghami et al., 2003; Beck et al., 2011) and it directly interacts with CNOT1, it could serve as a versatile platform to couple deadenylation with decapping and decay machineries in a broad variety of regulatory pathways.

A multitude of RNA-seq, CLIP-seq and ribosome profiling studies led to diverging conclusions on whether Ddx6-bound transcripts are committed to degradation or sequestered and stored in a translationally repressed state (Radhakrishnan et al., 2016; Courel et al., 2019). In *S. cerevisiae*, Ddx6 (Dhh1) preferentially binds and promotes the degradation of transcripts with suboptimal codons (Radhakrishnan et al., 2016). Conversely, in HEK293 cells Ddx6 represses the translation of suboptimal codon-containing transcripts that are AU-rich without affecting their stability, but promotes the degradation of GC-rich transcripts (Courel et al., 2019). Both during human embryonic stem cell differentiation (Freimer et al., 2018) and in *C. elegans* oogenesis (Boag et al., 2008), Ddx6 predominantly enables translational inhibition without affecting mRNA stability. The mode of silencing by Ddx6 can also change in the course of development; in early *Drosophila* embryo, Ddx6 (Me31B) binding is initially associated with reduced translational efficiency at 0–1 h post egg-laying, without impacting mRNA stability, but is correlated with reduced mRNA stability at later timepoints (1–3 h) (Wang et al., 2017). This transition is attributed to the reduced availabilities of Ddx6 (Me31B)’s main interacting partners 4E-T (Cup) and Lsm14 (Trailer Hitch) in the later developmental stages (Zavortink et al., 2020). The different conclusions drawn from these diverse studies suggest that the fate of Ddx6-bound mRNAs could vary depending on intrinsic mRNA features, but also, as with the other decapping activators discussed above, through availability and competitive interactions with decapping or translational factors.

## P Bodies: A Hub of Decapping and Decay Factors

Immunofluorescence and proteomics analyses in yeast and metazoans suggest that deadenylation, decapping, decay and translational repression factors co-localize in P bodies (Hubstenberger et al., 2017; Standart and Weil, 2018; Youn et al., 2018). Like stress granules, germ granules and several analogous structures, P bodies are membrane-less ribonucleoprotein (RNP) condensates which form through liquid-liquid phase separation (LLPS) (Courchaine et al., 2016). In contrast to canonical aqueous phase interactions, LLPS condensates are characterized by low-affinity, but multivalent interactions between RNA and proteins, especially with those encoding IDRs (Shin and Brangwynne, 2017).

Several studies support an important role for decapping factors in P body organization and composition. Loss of or point mutations in several decapping factors leads to abnormal, reduced or even loss of detectable P bodies *in vivo*, and some decapping factors are sufficient to induce LLPS *in vitro* (Luo et al., 2018; Standart and Weil, 2018). This is typically interpreted as a propensity to integrate/localize to existing P bodies *in vivo*, enhancing their stability, or even in *de novo* nucleation of new P body assemblies. Quantification of P body proteins and profiling of their mobility using FRAP (Fluorescence Recovery After Photobleaching) revealed that Dcp2 is the most concentrated and stable component of P bodies in *S. cerevisiae* (Xing et al., 2020). Dcp2 localization to P bodies was attributed to the N-terminal regulatory domain, its RNA-binding residues, and its interaction with Edc3, with mutations in these determinants resulting in the dispersal of Dcp2 in the cytoplasm of *S. cerevisiae* (Xing et al., 2020). The importance of the interaction with Edc3 agrees with findings from *in vitro* phase separation assays suggesting that a combination of recombinant *S. pombe* Edc3 or PatL1 (Pat1) with HLM-containing Dcp2 fragments can form LLPS condensates (Fromm et al., 2014; Lobel and Gross, 2020). Furthermore, RNA binding and self-dimerization of Edc3 also promote the formation of Dcp1/2-containing LLPS condensates *in vitro* (Schutz et al., 2017). Finally, the autoinhibitory motifs *in S. pombe* Dcp2 are also necessary for LLPS *in vitro* (Tibble et al., 2021), although whether these motifs promote or are required for P body localization *in vivo* remains to be investigated.

Ddx6 also plays a critical role in P body assembly, and its activities also affect the LLPS dynamics. Deletion of Ddx6 (Dhh1) results in the loss of up to 80% of Dcp2-containing P bodies in yeast and human cells (Ayache et al., 2015; Mugler et al., 2016). Mutations impairing ATP hydrolysis and helicase activities of Ddx6 (Dhh1) in *S. cerevisiae* favor the assembly of P bodies characterized by the Xrn1, Dcp1, Dcp2 and Edc3 markers (Mugler et al., 2016), presumably by locking the protein in an RNA-bound state. Conversely, the helicase activity of Ddx6 is necessary for P body formation in human induced pluripotent stem cells (Di Stefano et al., 2019) and in a human cancer cell line (Jangra et al., 2010). Thus, Ddx6 could drive P body assembly by remodelling RNA and promoting extensive interactions between other decapping proteins, likely in a cell-type-specific manner.

Interactions between different decapping activators can also influence the composition of P bodies. For example, the C-terminus of Edc4 contains an invariant arginine residue that is required for localization to P bodies in *Drosophila* (Jinek et al., 2008), and possibly in other species. In human cell lines, siRNA knockdown or point mutations of Edc4 significantly reduce the number of Dcp1-containing P bodies, suggesting that Edc4 promotes the recruitment of Dcp1 into P bodies (Seto et al., 2015; Mikuda et al., 2018). Another key determinant is the LSm domain of *Drosophila* Edc3 which is necessary and sufficient for its own localization to P bodies (Tritschler et al., 2007). Lastly, in both *S. cerevisiae* and human cells, the Mid and C domains of PatL1 are sufficient for P body localization (Pilkington and Parker, 2008), while this is mainly driven by the N-terminal Proline Rich Sequence in *Drosophila* PatL1 (Haas et al., 2010).

Due to the many combinations of IDR-mediated protein-protein and protein-RNA interactions possible among the decapping proteins detailed above, precisely discerning which factors and interactions underlie the assembly of P bodies remains a challenge. Another common difficulty in the interpretation of *in vivo* imaging results stems from the inconsistent choice of P body markers among studies. Use of only one or a few P body markers can confound loss of punctate imaging for a particular P body component with the complete loss of P body assembly.

### Known and Suspected Functions for P Bodies in Decapping and Decay

Several observations support the possibility that P bodies represent active sites for mRNA decapping and decay. Firstly, abolishing decapping or decay activities via genetic depletion of Dcp2 or Xrn1 enlarges P bodies in both *S. cerevisiae* and *Drosophila* (Sheth and Parker, 2003). Secondly, kinetic measurements of *S. pombe* Dcp2 activity *in vitro* demonstrated that Edc3 alleviates Dcp2 autoinhibition and promotes its activity in LLPS condensates (Tibble et al., 2021). Similarly, deadenylation of reporter transcripts is enhanced in phase-separated granules *in vitro* (Sheu-Gruttadauria and MacRae, 2018). A simplistic view is that the convergence of deadenylation, decapping and decay factors inside P bodies might ensure a coupling between deadenylation and decay and enhance their kinetics, thereby leading to robust mRNA turnover.

In contrast with this model, several observations suggest that decapping and decay can, and do also occur outside of P bodies. Firstly, mutations that abolish visible Edc4- and 4E-T (Trailer Hitch)-positive P bodies in *Drosophila* S2 cells had no impact on the degradation of reporter mRNAs (Eulalio et al., 2007a). Secondly, Edc1 can activate decapping in the cytoplasm as well as in LLPS condensates *in vitro* (Tibble et al., 2021). Thirdly, quantification of cytoplasmic versus P body–localized pool of decapping proteins in *S. cerevisiae* suggests that only a small fraction (∼10%) of decapping activators localizes to P bodies, with the exception of Dcp2 for which ∼30% of the total pool is concentrated in P bodies (Xing et al., 2020). Therefore, while P bodies may be a possible site for enhanced decay, several lines of independent evidence indicate that they are not required and certainly not the sole site for mRNA decapping and decay. It is possible that P bodies can serve to sequester specific sets of mRNAs away from the cytoplasm, to be degraded upon regulated cellular or environmental cues. In support of this, comparative RNA-seq of P bodies purified from *S. cerevisiae* revealed that different sets of mRNAs are enriched in P bodies under different stress conditions (Wang et al., 2018). Metabolic labelling showed that some of these P body–localized transcripts are destabilized, although this study could not conclusively infer whether their destabilization occurs inside or outside of P bodies (Wang et al., 2018). Since components of the 3′-to-5′ decay pathway are depleted from P bodies (Hubstenberger et al., 2017; Youn et al., 2018), it is also possible that P body localization may sort specific mRNAs toward a particular decay route.

Although the aforementioned evidence supports a function for P bodies in RNA decapping and decay, substantial evidence instead indicates that they can serve as a storage compartment for translationally repressed mRNAs. It was noticed early on that P bodies increase in size upon inhibition of translation and disassemble upon translational reactivation, in both yeast and *Drosophila* cells (Brengues et al., 2005; Eulalio et al., 2007a). More recently, ribosome profiling and RNA-seq of purified Lsm14-positive P bodies from HEK293 cells indicate an enrichment of transcripts that are poorly translated (Hubstenberger et al., 2017). Furthermore, a detailed single-molecule resolution kinetic study in HEK293 cells did not detect any difference in mRNA decay rate inside or outside of Ddx6-containing P bodies (Wilbertz et al., 2019). The lack of observed differences in mRNA decay rates may be in part due to the selective purification of Ddx6- or Lsm14-containing P bodies in the latter two studies. As discussed above, in some conditions, both Ddx6 and Lsm14 can repress translation without affecting mRNA stability. It will be interesting to compare this observation with the mRNA profile of purified P bodies obtained from selective enrichment of Dcp2 or Edc1-4.

Notwithstanding the above, the diversity of proteins and interactions upon which P bodies are scaffolded could reflect a functional heterogeneity across P body foci, with some favoring or dedicated to translational repression and others biochemically geared towards decapping and decay. From a functional standpoint, a few reports suggested diverging roles for P bodies in distinct cellular states. On one hand, P bodies promote differentiation of pluripotent stem cells, as well as neural and intestinal progenitors in human cells (Di Stefano et al., 2019). Conversely, they maintain the pluripotency of mesenchymal stem cells in human cells (Di Stefano et al., 2019) and *Drosophila* (Buddika et al., 2022). It stands to reason that intrinsic features of targeted mRNAs, interactions with RNA-binding proteins, or spatiotemporal expression of P body components may alter P body composition and function. Detailing the heterogeneity of P bodies across cell types, but also within a defined cell state, may prove useful in resolving the apparent functional divergence and specificity of the roles for these pervasive foci.

## Current Models of Decapping Activation

**FIGURE 4:**
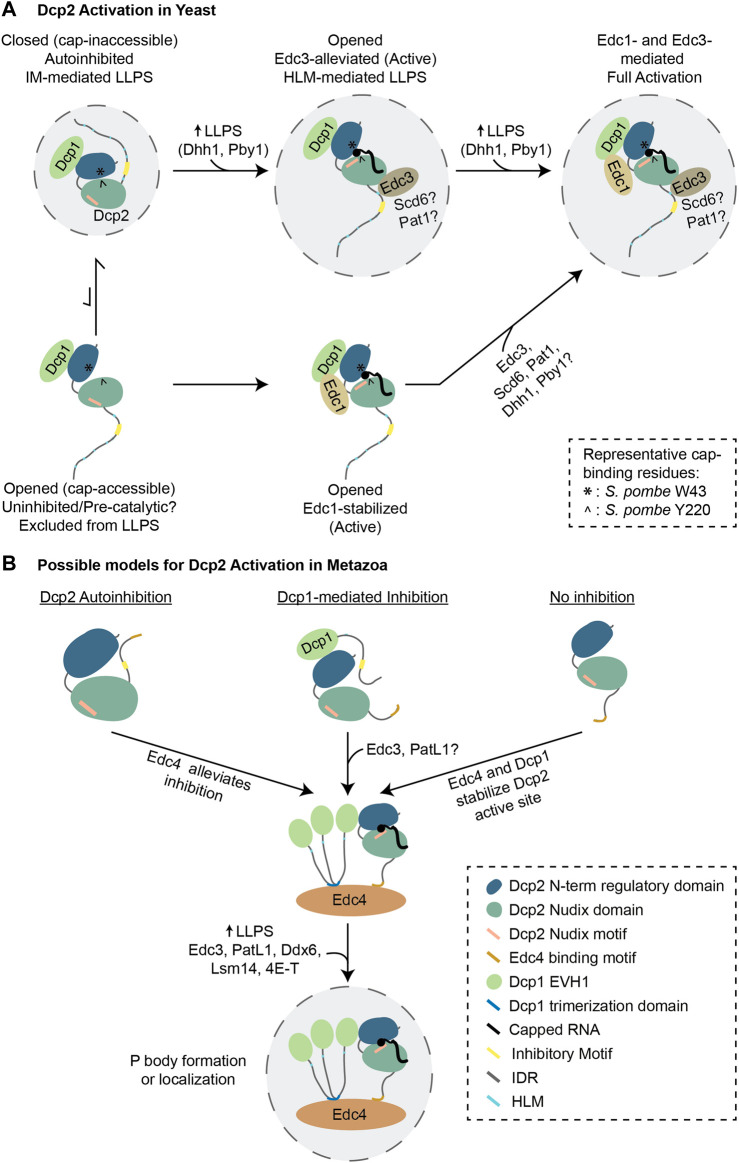
Models of decapping activation in yeast and metazoans. **(A)** The current model of Dcp2 activation from *S. pombe* is depicted. Dcp1/2 predominantly exists in an autoinhibited state that self-assemble into LLPS condensates and maintained by the interaction between W49 and Y220. Edc3 binding to Dcp2 HLM likely reorganizes the C-terminal IDR, allowing the formation of an active site in which W49 and Y220 interact with the cap, and activating Dcp2 inside LLPS condensates. On its own, Edc1 may stabilize the opened/active conformation of Dcp2 or consolidate the formation of active site from a pre-catalytic conformation from outside of LLPS condensates. Edc1 can also stabilize the Edc3-alleviated conformation in LLPS condensates, contributing to full activation of Dcp2. **(B)** Hypothetical model of metazoan Dcp2 activation. It is currently unknown whether or not metazoan Dcp1/2 is regulated through autoinhibition. Since metazoan Edc4 promotes the interaction between metazoan Dcp1 and Dcp2, it may help to alleviate autoinhibition or promote the formation of active site on Dcp2. Other decapping activators might enable decapping by promoting phase separation and Dcp2 localization to LLPS condensates. Abbreviations: IDR = Intrinsically Disordered Region; HLM = Helical Leucine-rich Motif.

Having discussed the structure and functions of decapping proteins and their convergence in P bodies, we will next describe current models of decapping activation (Figure 4). Our understanding of Dcp1/2 activation has been largely driven through structural work on the yeast Dcp1/2 holoenzyme on its own or bound to activators and cap analogs. Comparison of such structures proved key in revealing the multiple conformations of the holoenzyme and allowing consolidation into a dynamic model (She et al., 2008; Floor et al., 2010; Floor et al., 2012; Charenton et al., 2016; Mugridge et al., 2016; Valkov et al., 2016; Wurm et al., 2017; Mugridge et al., 2018b) (Figure 4A).

Current evidence strongly supports that decapping activation is largely due to conformational rearrangements within the Dcp1/2 complex. In yeast, the formation of a catalytically competent cap-binding pocket in Dcp2 is determined by the orientation of the N-terminal regulatory domain relative to the Nudix hydrolase domain (She et al., 2008; Floor et al., 2010; Floor et al., 2012; Charenton et al., 2016; Mugridge et al., 2016; Valkov et al., 2016; Wurm et al., 2017; Mugridge et al., 2018b). On its own, Dcp2 rapidly transits between opened (cap-accessible) and closed (cap-inaccessible) conformations at similar rates, but its interaction with Dcp1 strongly biases the equilibrium towards the closed conformation (Wurm et al., 2017) (Figure 4A, top left). In the closed conformation, the N-terminal regulatory domain is positioned on top of the Nudix hydrolase domain, and the essential cap-binding residues (*S. pombe* W43 and Y220) are buried (She et al., 2008; Charenton et al., 2016; Mugridge et al., 2016; Wurm et al., 2017; Mugridge et al., 2018b). The N-terminal regulatory domain of Dcp2 also lies on top of the Box B motif, thereby occluding the RNA-binding channel (Charenton et al., 2016). Furthermore, the autoinhibitory effect carried by the C-terminal IDR of Dcp2 is consistent with the closed conformation, and a point mutation in the cap-binding Y220 residue in *S. pombe* effectively alleviates autoinhibition (Paquette et al., 2018). Intriguingly, recent *in vitro* studies showed that recombinant Dcp1 and Dcp2 fragments that encompass the inhibitory motifs self-assemble into LLPS condensates where they remain inactive, and indeed such phase separation is dependent on the inhibitory motifs themselves (Tibble et al., 2021). This suggests that in the absence of decapping activators, LLPS may create a repressive environment for the Dcp1/2 complex (Tibble et al., 2021).

Decapping activators promote Dcp1/2 activity via several distinct mechanisms. The binding of Edc3 to Dcp2 reconfigures the cap-binding residues and folding of the Box B motif to accommodate RNA binding (Charenton et al., 2016). Edc3 also alleviates Dcp2 autoinhibition, by remodelling the inhibitory motifs in the Dcp2 C-terminal IDR (Paquette et al., 2018). This mode of activation is coupled to phase separation, and is dependent on the HLMs of Dcp2, instead of the inhibitory motifs (Tibble et al., 2021) (Figure 4A, top middle).

Decapping can also be enhanced through stabilization of the active conformation of Dcp1/2. This is the case with Edc1, for example, which on its own does not alleviate the autoinhibition of Dcp1/2 (Paquette et al., 2018). Instead, Edc1 makes extensive contacts with Dcp1 and stabilizes the cap-binding groove formed by the N-terminal and the Nudix hydrolase domains of Dcp2 (Mugridge et al., 2016; Wurm et al., 2017; Mugridge et al., 2018b). Since Edc1 can promote decapping from inside or outside LLPS condensates (Tibble et al., 2021), it may enhance the activity of a rarer subset of opened but poorly active Dcp1/2 conformations outside of LLPS condensates (Figure 4A, bottom left to middle). Lastly, Edc1 can cooperate with Edc3 inside of LLPS condensates by binding to the Edc3-derepressed pool of Dcp1/2, resulting in a faster decapping rate compared to the activation by Edc1 or Edc3 alone (Mugridge et al., 2018b; Tibble et al., 2021) (Figure 4A, top right).

These models of decapping activation provide a conceptual framework to understand how other decapping activators may function. How the yeast Lsm14 (Scd6) and PatL1 (Pat1) decapping activators influence kinetics along the continuum of Dcp1/2 conformations remains to be investigated. Since the LSm domain of *S. cerevisiae* Lsm14 (Scd6) can bind Dcp2 HLMs as does Edc3 (Fromm et al., 2012), it may effect Dcp1/2 holoenzyme activation through a similar mechanism. However, one could expect that the presence of RGG motif(s) in lieu of the YjeF-N homodimerization domain (Albrecht and Lengauer, 2004) may yield some differences in how Lsm14 (Scd6) and Edc3 promote LLPS. *S. pombe* PatL1 (Pat1) was predicted to alleviate autoinhibition of Dcp1/2 as it could promote decapping activity in the presence of inhibitory motifs, although at the apparent cost of lowering the affinity for RNA (Lobel et al., 2019). Furthermore, the interaction of PatL1 (Pat1) with the HLMs of Dcp2 in yeast may alter the conformation of Dcp2 C-terminal IDR to alleviate autoinhibition (Charenton et al., 2017; Lobel et al., 2019). A crystal structure of PatL1 (Pat1) bound to Dcp1 and Dcp2 fragments that contain the HLMs and inhibitory motifs would help shed light on how autoinhibition is alleviated, and whether it involves mechanisms that are also leveraged by Edc1 and Edc3. As with Edc3, Ddx6 and Pby1 may also couple decapping enhancement with LLPS, but their precise mechanisms remain to be investigated.

The prevailing model of Dcp1/2 activation in metazoans was put forth by the Izaurralde lab, and posits Edc4 as a scaffold for the interactions between Dcp2, Dcp1 trimers and Xrn1 (Chang et al., 2014) (Figure 4B). Beyond this scaffolding function, however, whether and how Edc4 influences the conformation of the decapping holoenzyme is unknown, and cannot confidently be extrapolated from the yeast model where Edc4 is not conserved or recognizable. Additional major differences with metazoans include a significant difference in length of Dcp2 IDR, and the lack of recognizable inhibitory elements. Thus, a critical question to refine the mechanism of metazoan Dcp2 activation is whether it is subjected to autoinhibition. A possibility inferred from the increased length of Dcp1 C-terminal IDR is that Dcp1-encoded regulatory elements might inhibit Dcp2, and that this inhibition is in turn alleviated by Edc4 and/or other decapping activators. Alternatively, metazoan Dcp2 might simply not be robustly self-inhibited. The full activation of the catalytic site may instead require a conformational change induced by Dcp1 and the decapping activators. Complementation experiments to assess the decapping and decay of mRNA reporters in the presence of Dcp2 and Dcp1 fragments could be informative. As with our evolving understanding of the yeast Dcp1/2 activation, high-resolution structures of the active and inactive conformations of metazoan Dcp1/2 in complex with the decapping activators, would be key to this interesting problem.

## Perspective and Emerging Questions

Nearly 50 years since the first decapping activity was detected, countless discoveries across species and experimental systems have revealed key players of decapping and much of their mechanisms of action. Still, critical and long-standing questions persist, and novel emerging questions are likely to justify revisions to the model of how mRNAs are decapped.

The developmental regulation of mRNA decapping and decay will likely reveal unexpected twists on how subsets of transcripts meet their fate. A striking paradigm is the maternal-to-zygotic transition (MZT), where 25–60% of maternally deposited transcripts are degraded in all animal species (Vastenhouw et al., 2019). In *D. rerio* embryos, MZT involves deadenylation largely instigated by the maternally contributed miR-430 (Giraldez et al., 2006), wherein 3′ terminal uridylation triggers the degradation of deadenylated mRNAs (Chang et al., 2018). While Dcp2 has been implicated in the decay of over 1,000 maternal transcripts during *D. rerio* MZT (Mishima and Tomari, 2017), the contributions of decapping scaffolds and activators remain to be studied. Genome-wide analysis of 3′-untranslated regions suggests that combinations of *cis* elements and their cognate RNA-binding proteins can be used to predict the susceptibility of transcripts to decay (Vejnar et al., 2019). Furthermore, m^6^A (N^6^-methyladenosine) RNA modification was shown to promote mRNA degradation during MZT (Zhao et al., 2017), while m^5^C (5-methylcytosine) has the opposite effect (Yang et al., 2019). How any of these determinants intersect with the Dcp1/2 holoenzyme and its activators is an open, but important question.

How viruses manipulate mRNA cap metabolism is a promising area for advances on decapping regulation in health and disease. A great diversity of viruses leverage or manipulate mRNA decay machineries to favor viral RNA translation and evade host immune response (Guo et al., 2018). Most viruses that globally destabilize the host mRNAs encode viral endonucleases, thereby bypassing host deadenylation, decapping and decay machineries (Abernathy and Glaunsinger, 2015). Some negative-strand RNA viruses such as the Bunyaviruses evolved a “cap snatching” mechanism, whereby a viral endonuclease competes with Dcp2 to cleave mRNAs at 8–10 nucleotides downstream of the cap so that the resulting fragment can be used to initiate the transcription of m^7^G-capped viral RNA (Hopkins et al., 2013). Other viruses such as Poxviruses, African Swine Fever Virus and Mimivirus encode their own decapping enzymes and effectively compete with host decapping machineries (Quintas et al., 2017; Cantu et al., 2020; Kago and Parrish, 2021). For example, Vaccinia Virus D9 and D10 decapping enzymes synergistically promote viral replication by dampening the host innate immunity through suppression of response to viral double-stranded RNA (Burgess and Mohr, 2015; Liu et al., 2015). A recent high-resolution crystal structure of D9 revealed that its Nudix fold is intertwined by three-helical bundle (Peters et al., 2021), possibly imparting different specificity or kinetic advantage against the host Dcp2. Such functional and kinetic viral decapping paradigms also bear the promise of identifying novel opportunities for antiviral therapies.

Structurally, three of the most pressing questions are how the metazoan Dcp1/2 holoenzyme is catalytically activated or de-repressed, what the mechanistic contribution of IDR regions is in the two proteins and regulatory elements they may encode, and lastly what interplay they may have with decapping co-activators such as Edc4. From our perspective, structures of Dcp1 and Dcp2 that would include their IDRs will be critical to developing a comprehensive model of Dcp2 activation. Difficulties in crystallizing or resolving IDR sequences represent a major barrier towards resolving these issues. Adaptations of structural prediction tools such as AlphaFold (Jumper et al., 2021) may provide a steppingstone to develop credible models of the organization of low-complexity determinants, and in allowing the identification of structurally informed elements that can be tested through mutational analyses.

The importance of P bodies for decapping *in vivo* remains controversial. This is understandable considering the prevailing and superficially conflicting models derived from independent studies in a variety of experimental models. Ultimately, a definitive examination of decapping and decay kinetics in individual P bodies, *in vitro* and *in vivo,* would provide definitive answers to this important question. In the meantime, the recent *in vitro* reconstitution of decapping LLPS using a limited subset of P body constituents (Dcp2, Edc1 and Edc3) has provided the first biochemical evidence that activation of decapping by at least one activator (Edc3) is coupled to LLPS (Tibble et al., 2021). Cell-free systems may provide a suitable experimental approach to bridge *in vitro* studies of LLPS using recombinant proteins with *in vivo* analyses of P bodies. Recently, stress granules and nucleolus formation were successfully recapitulated in mammalian cell lysates, seeded with nucleator proteins G3BP1 and NPM1 respectively (Freibaum et al., 2021). Coupling such a cell-free system with quantitative single-molecule imaging would provide a powerful perspective on decapping kinetics, inside or outside of P bodies. The effect of each decapping activator on P body formation and decapping enhancement can also be systematically studied using cellular lysates obtained from various knockouts of decapping activators.

Lastly, our understanding of the integration of physiological and environmental cues on the decapping machinery and P bodies through signalling pathways remain far from comprehensive. A few examples have recently emerged. For example, phosphorylation of Edc3 and Edc4 by the Pim1/3 kinase and the IκB kinase (IKK), respectively, promote their localization to P bodies in human cells (Mikuda et al., 2018; Bearss et al., 2021). Similarly, ubiquitination and phosphorylation of Dcp1 by the TRAF6-JNK signaling pathway upon cytokine induction is important for Dcp1 to localize to P bodies (Tenekeci et al., 2016).

Considering the critical role for the 5′-cap over the life cycle of transcripts and the decisive demise step that is mRNA decapping, it seems unavoidable that the Dcp1/2 holoenzyme activity is not only kept under tight check, but that it is also closely tuned with a cell’s developmental and metabolic state.

## Further Readings

We intended this review to complement other review articles that have discussed the processes and determinants upstream of decapping (Mayya and Duchaine, 2019), functional implications of decapping (Borbolis and Syntichaki, 2021), broader themes in decapping-dependent mRNA decay (Li and Kiledjian, 2010; Valkov et al., 2016; Mugridge et al., 2018a), the detailed structure and enzymology of the Dcp1/2 complex (Charenton and Graille, 2018; Kramer and McLennan, 2019), and provide an updated view on the structure and function of decapping activators (Jonas and Izaurralde, 2013). We refer readers to other articles for a more in-depth discussion on related topics that we could not cover in detail, such as the diverse mRNA cap modifications and their processing (Cougot et al., 2004; Galloway and Cowling, 2019; Pelletier et al., 2021), connections between translation and mRNA decay (Huntzinger and Izaurralde, 2011; Hanson and Coller, 2018; Heck and Wilusz, 2018), non-Dcp2 Nudix hydrolases (Srouji et al., 2017; Kiledjian, 2018), nonsense-mediated decay (NMD) (Jaffrey and Wilkinson, 2018), endonuclease-mediated decay (Schoenberg, 2011), mRNA surveillance mechanisms (Wolin and Maquat, 2019) and nuclear RNA decay (Schmid and Jensen, 2018).
